# Consolidation chemotherapy during neoadjuvant chemoradiation (CRT) for distal rectal cancer leads to sustained decrease in tumor metabolism when compared to standard CRT regimen

**DOI:** 10.1186/s13014-016-0598-6

**Published:** 2016-02-24

**Authors:** Angelita Habr-Gama, Rodrigo O. Perez, Guilherme P. São Julião, Igor Proscurshim, Laura M. Fernandez, Marleny N. Figueiredo, Joaquim Gama-Rodrigues, Carlos A. Buchpiguel

**Affiliations:** Angelita & Joaquim Gama Institute, Rua Manoel da Nóbrega 1564, São Paulo, SP Brazil; University of São Paulo School of Medicine Colorectal Surgery Division, São Paulo, Brazil; University of São Paulo School of Medicine, São Paulo, Brazil; Ludwig Institute for Cancer Research São Paulo Branch, São Paulo, Brazil; University of São Paulo School of Medicine Nuclear Imaging Division, São Paulo, Brazil

**Keywords:** Rectal cancer, Neoadjuvant chemoradiation, Consolidation chemotherapy, PET/CT, Response assessment

## Abstract

**Background:**

Neoadjuvant CRT may lead to significant tumor regression in patients with rectal cancer. Different CRT regimens with consolidation chemotherapy may lead to increased rates of complete tumor regression. The purpose of this study was to understand tumor metabolic activity following two different neoadjuvant CRT regimens using sequential PET/CT imaging in two different intervals following RT.

**Methods:**

Patients with cT2-4 N0-2 M0 rectal cancer treated by standard CRT (54Gy and 2 cycles of 5FU-based chemotherapy) or extended CRT (54Gy and 6 cycles of 5FU-based chemotherapy) underwent sequential PET/CT imaging at baseline, 6 weeks and 12 weeks from radiation completion.

**Results:**

99 patients undergoing standard CRT were compared to 12 patients undergoing CRT with consolidation chemotherapy. Patients treated with consolidation CRT had increased rates of complete clinical or pathological response (66 % vs. 23 %; *p* < 0.001). SUVmax variation between baseline and 6 weeks (88 % vs. 63 %; *p* < 0.001) and between baseline and 12 weeks (90 % vs. 57 %; *p* < 0.001) were significantly more pronounced among patients undergoing extended CRT with consolidation chemotherapy. An increase in SUVmax between 6 and 12 weeks was observed in 51 % of patients undergoing standard and 18 % of patients undergoing consolidation CRT (*p* = 0.04).

**Conclusions:**

Most of the reduction in tumor metabolism after neoadjuvant CRT occurs within the first 6 weeks from RT completion. In patients undergoing CRT with consolidation chemotherapy, tumors are less likely to regain metabolic activity between 6 and 12 weeks. Therefore, assessment of tumor response may be safely postponed to 12 weeks in patients undergoing extended CRT with consolidation chemotherapy.

**Trial registration:**

NCT00254683

## Background

Neoadjuvant chemoradiation therapy (CRT) is now considered one of the preferred treatment strategies for distal rectal cancer [1]. In addition to the potential benefits in improved local disease control, neoadjuvant CRT may result in significant tumor downsizing and tumor downstaging [2]. Ultimately, complete tumor regression may develop with no residual cancer cells found at pathology following radical surgery [3, 4]. These patients with complete pathological response (pCR) are associated with improved oncological outcomes. Accurate identification of these patients could potentially spare them from major radical proctectomy and its considerable postoperative morbidity, mortality and disturbances in urinary, fecal and sexual functions [5]. Both full-thickness local excision and observation alone without immediate surgery have been suggested as acceptable alternative organ-preserving treatment strategies for these patients [5–8].

Ultimately, alternative treatment regimens that could improve or maximize complete response (CR) rates could have a significant impact on oncological outcomes and need for radical surgery in patients with rectal cancer. Recently, the incorporation of additional cycles of 5-Fluorouracil (5FU) based chemotherapy to standard long-course CRT regimens has resulted in considerably higher CR rates allowing avoidance of surgery in more than half of patients with stage I-III rectal cancer [9].

The role of molecular imaging using ^18^F-fluorodeoxyglucose (FDG) positron emission tomography/computerized tomography (PET/CT) has been extensively investigated in the setting of neoadjuvant CRT to predict and assess tumor response in patients with rectal cancer [10–14]. Besides the qualitative information provided by this imaging modality, PET/CT allows objective estimation of tumor metabolism based on standard uptake values (SUV). In fact, series of published reports have consistently suggested that greater decreases in tumor metabolism at different intervals from CRT completion are associated with improved rates of CR and overall outcomes [13, 15–18]. In addition, it has been observed that a significant proportion of patients develop recuperation of tumor metabolism following standard CRT regimens between 6 and 12 weeks [19]. This observation could potentially reflect significant tumor cell death between baseline and 6 weeks followed by repopulation of cancer cells between 6 and 12 weeks from CRT completion.

Therefore, we decided to investigate tumor metabolism with the use of FDG PET/CT imaging after extended CRT using additional (consolidation) cycles of chemotherapy during radiotherapy (RT) and the resting period in comparison to standard CRT (2 cycles of concomitant 5FU-based chemotherapy). One would expect that regimens with improved rates of complete response would have more pronounced decreases in tumor metabolism and decreased risk for recuperation of tumor metabolism after 6 weeks from RT completion.

## Methods

Consecutive patients with non-metastatic rectal adenocarcinoma located up to 7 cm from the anal verge (assessed by rigid proctoscopy) were eligible for the study after local IRB-approval (CAPPESQ 717/05; University of São Paulo School of Medicine). Informed consent was obtained from all individual participants included in the study. Baseline staging and assessment included full physical and digital rectal examination (DRE), rigid proctoscopy, endorectal ultrasound (ERUS) or high-resolution magnetic resonance (MR). Considering all patients underwent PET/CT, imaging additional regular chest and abdominal computerized tomography (CT) scans were omitted for systemic staging. All patients with cT2-4 N0-2 M0 were included in the study unless refusal to sign informed consent, refusal to undergo CRT, pregnancy or age <18 years were present. There was no difference in policy for the indication to neoadjuvant CRT between the two cohorts.

### Neoadjuvant CRT

#### Standard CRT regimen - NCT00254683

Patients undergoing standard CRT have been previously described from the prospective trial registered under NCT00254683 that recruited patients between 2005 and 2009 at the University of São Paulo School of Medicine – Colorectal Surgery Division after IRB approval [14]. Briefly, all patients underwent 54Gy of radiation and concomitant chemotherapy (5FU/leucovorin; 5FU 425 mg/m^2^/d; folinic acid 20 mg/m^2^/d) being delivered in the first and last 5 days of radiation (2 cycles of chemotherapy). A total of 45Gy in daily doses of 180 cGy was delivered to the pelvis and an additional boost of 9Gy was delivered to the primary tumor [14].

#### Consolidation CRT regimen

Consecutive patients undergoing extended CRT between 2012 and 2013 at the Angelita & Joaquim Gama Institute received 54Gy of radiation using similar fractionation and 5FU-based chemotherapy (5FU/leucovorin) as described elsewhere and IRB approval [20]. These patients received a total of 6 cycles of chemotherapy (3 consecutive days each with bolus 5FU 450 mg/m^2^ and a fixed dose of 50 mg of leucovorin) being delivered during the radiation therapy period (3 cycles) and additional 3 cycles after radiation completion (resting period), instead of the 2 usual cycles in the beginning and end of RT.

### Assessment of response

In both groups of patients, clinical assessment of tumor response was performed uniformly at 12 weeks from RT completion by two experienced colorectal surgeons using identical assessment tools used at baseline: Physical examination, DRE, rigid proctoscopy, ERUS or pelvic MR. Patients with clinical and radiological evidence of complete clinical response (cCR) were not immediately operated on and were enrolled in a Watch & Wait surveillance protocol as described elsewhere [21]. Patients with incomplete clinical response were referred to immediate radical surgery. Surgeons responsible for the assessment of response were unaware of PET/CT information at the time of assessment. Clinical management decision did not take PET/CT information into consideration unless unsuspected metastatic disease was detected by any of the 3 studies performed by each patient.

Follow-up of patients with complete clinical response managed non-operatively was performed with rigid proctoscopy, CEA and DRE every 1–2 months for the first year and every 3 months thereafter (up to 3 years of follow-up). After 3 years, patients were assessed every 6 months until 5 years and yearly thereafter. Radiological imaging for the pelvis (MR) and distant metastases (CT scans) were performed every 6 months. Patients undergoing radical surgery were followed every 3 months for the first 2 years, every 6 months between 2–4 years and yearly thereafter.

### PET/CT

In both groups (Standard and Consolidation CRT), all patients underwent baseline PET/CT prior to CRT. Following CRT completion, PET/CT was performed at 6 weeks and at 12 weeks after radiotherapy completion. All PET/CT scans were obtained after IV injection of 370 MBq of 2-fluorine-18-fluoro-2-deoxy-D-glucose (FDG) under a 6–12 h fasting period. Fasting serum glucose levels were measured 10–15 min before FDG injection according to standardized protocol. Data were acquired using a dedicated LSO-PET/CT scanner (Biograph 16, SIEMENS, IL) as previously described [14].

Whole body imaging was carried out in all patients covering from the base of skull to the proximal portion of thighs. Imaging was qualitatively interpreted by a single experienced observer (nuclear radiologist expert) who was unaware of the patients’ clinical-assessment status at baseline or following CRT.

SUVmax were recorded for primary tumors in all studies (baseline, 6-week and 12-week).

**Fig. 1:**
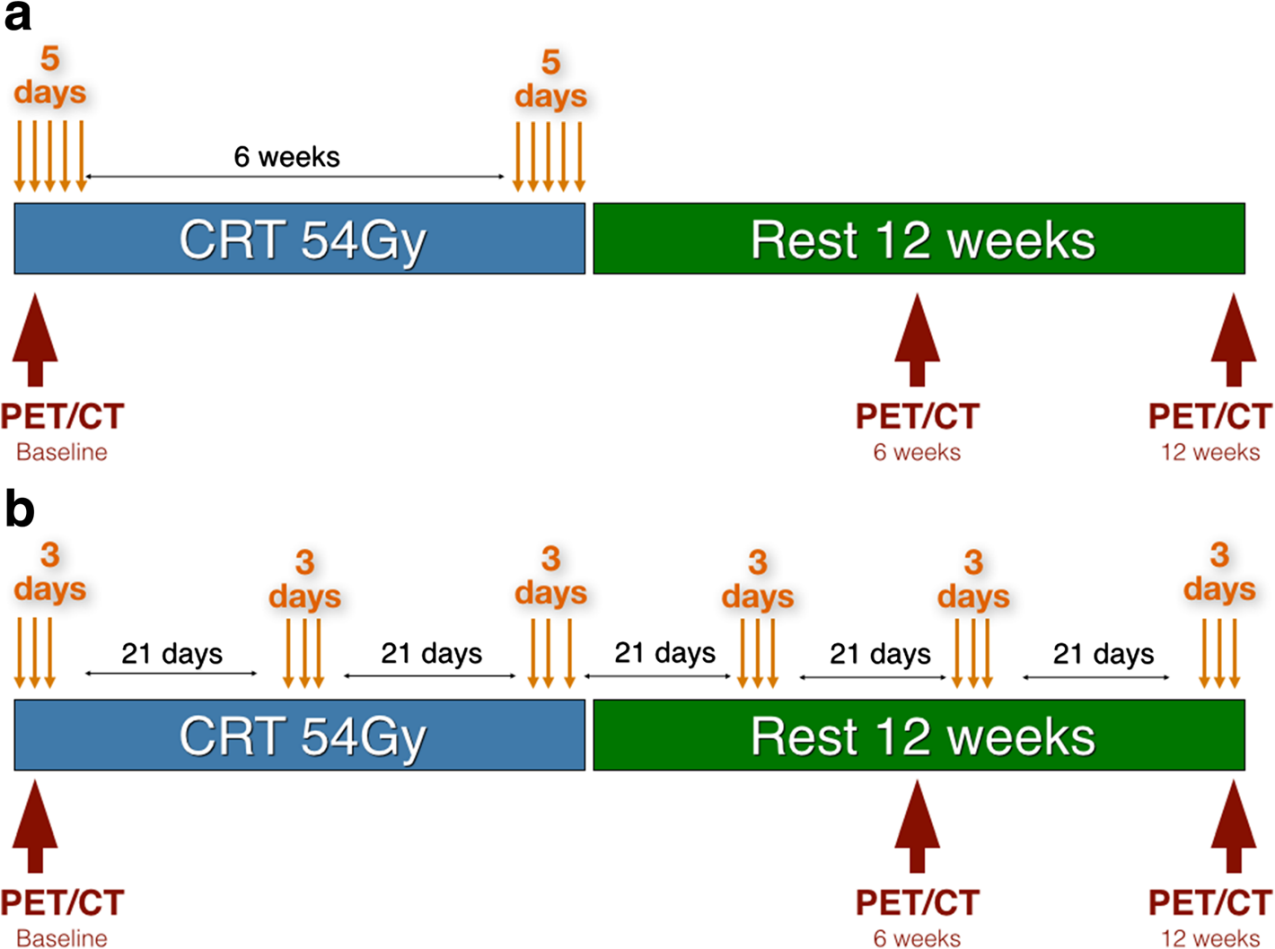
CRT regimens and PET/CT timing during treatment. **a** Standard CRT regimen – 54Gy of radiation associated to 2 cycles of 5FU based chemotherapy; **b** Consolidation CRT regimen – 54Gy of radiation associated to 6 cycles of 5FU based chemotherapy

PET/CT information was compared between patients undergoing standard and extended CRT (with consolidation chemotherapy). (Fig. 1).

### Statistical analysis

Statistical analysis was performed using SPSS V13.0 (Spss Inc, Chicago Il). Numerical variables were analysed by student t test and categorical variables were evaluated using Chi square test. Differences were considered statistically significant for *p* < 0.05.

## Results

Clinical and radiological outcomes of the 99 patients undergoing standard CRT enrolled in the prospective study are available elsewhere [14]. Briefly, of the 99 patients, 16 developed cCR (16 %) and 7 had pCR (7 %) after radical surgery (23 % overall CR rate).

**Fig. 2:**
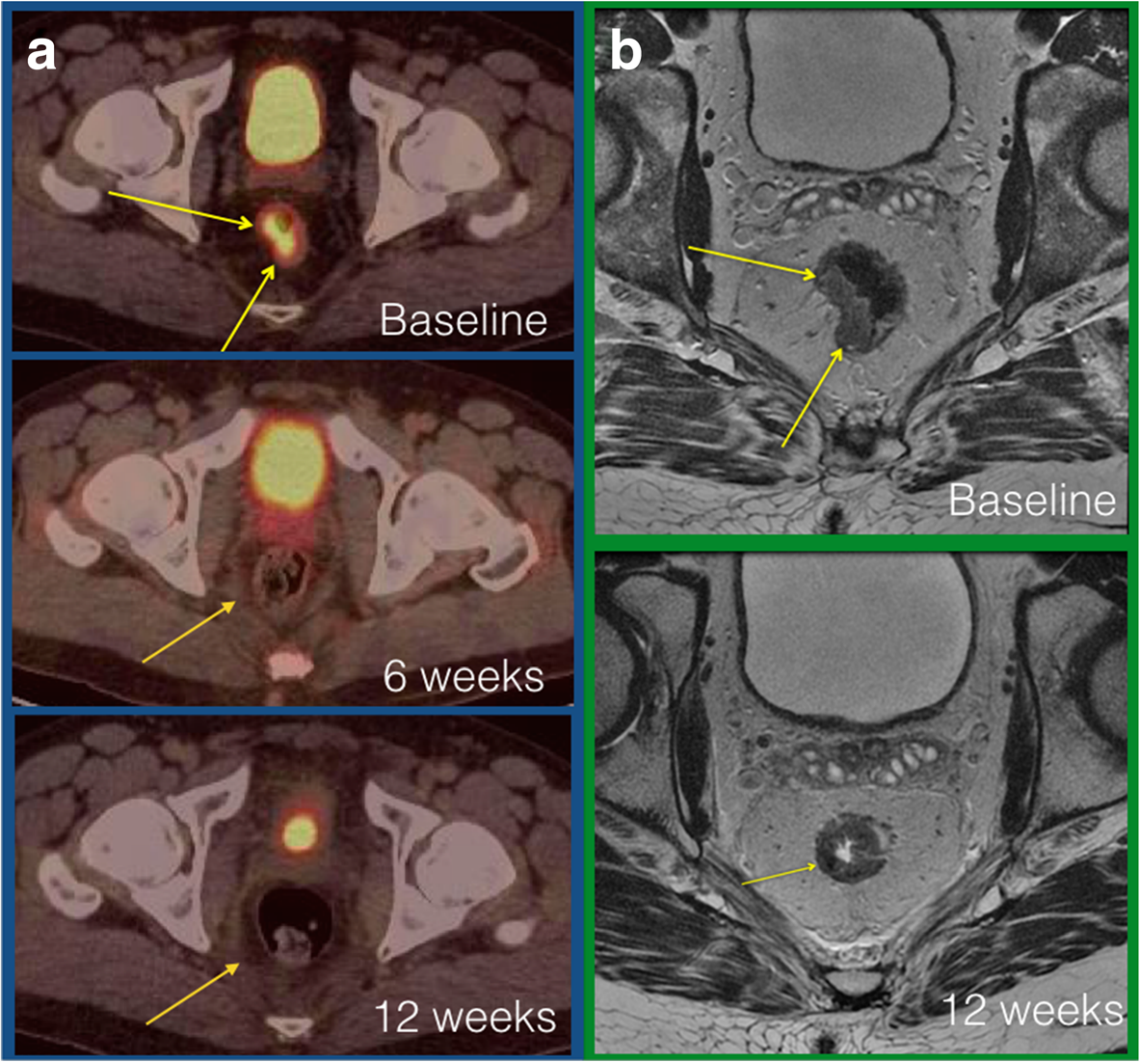
**a** PET/CT imaging (baseline / 6 weeks / 12 weeks) in a patient that developed complete clinical response; **b** MR imaging from the same patient at baseline and at 12 weeks after CRT

Overall, 12 patients underwent extended CRT with consolidation chemotherapy at the Angelita & Joaquim Gama Institute between 2012 and 2013. Of these, 7 patients developed cCR (58 %) and 1 had pCR (8 %) after this treatment strategy. Patient’s characteristics and baseline tumor features are available in Table 1. (Fig. 2).

**Table 1:** Patient’s characteristics and baseline tumor features

		Standard CRT	Extended CRT	*p*
N		99	12	
Gender (M-F)		47 – 52 (47.5 – 52.5)	7 – 5 (58.3 – 41.7)	0.48
Age (years)		60.3 ± 12.7	58.6 ± 8.4	0.53
Tumor size (mm)		43.2 ± 11.6	43.3 ± 6.5	0.96
Distance anal verge (cm)		3.9 ± 2.0	4.6 ± 1.3	0.13
Initial Staging				
cT	2	6 (6.1 %)	5 (41.7 %)	
	3	87 (87.9 %)	7 (58.3 %)	
	4	6 (6.1 %)	0 (0.0 %)	0.001
cN	positive	43 (43.4 %)	8 (66.7 %)	0.11
cUICC	I	6 (6.1 %)	3 (25.0 %)	
	II	50 (50.5 %)	1 (8.3 %)	
	III	43 (43.4 %)	8 (66.7 %)	0.006
TRG 3 or 4		22 (30.1 %)	3 (75.0 %)	0.09
CR (cCR or pCR)		23 (23.2 %)	8 (66.7 %)	0.004
Final tumor size (mm)		40.6 ± 22.2	26.4 ± 7.9	0.01
Final Staging				
UICC	CCR	16 (16.1 %)	7 (58.4 %)	
	pCR	7 (7.1 %)	1 (8.3 %)	
	I	14 (14.1 %)	1 (8.3 %)	
	II	30 (30.3 %)	0 (0.0 %)	
	III	19 (19.2 %)	2 (16.7 %)	0.004

Patients undergoing standard CRT had significantly more locally advanced disease when compared to consolidation CRT group in terms of cT status (cT3: 88 % vs 58 %; *p* < 0.001). Also, patients were less likely to develop complete pathological response or complete clinical response (23 % vs. 66 %; *p* = 0.004) after standard CRT.

### SUVmax

Baseline SUVmax was significantly higher among patients in the standard CRT group when compared to consolidation CRT group (21.5 vs. 14.8; *p* = 0.004). SUVmax was also consistently higher in the standard CRT group at 6 weeks and 12 weeks from CRT (Table 2).

**Table 2:** PET/CT characteristics according to neoadjuvant CRT regimen

	Standard CRT	Extended CRT	*p*
Baseline SUVmax	21.5 ± 11.0	14.8 ± 5.9	0.004
6 weeks SUVmax	7.3 ± 6.3	1.9 ± 1.8	0.001
12 weeks SUVmax	8.4 ± 7.4	1.3 ± 1.9	0.001
Complete Response	18 (18.2 %)	8 (66.7 %)	0.001
∆ %SUVmax variation (6 weeks - baseline)	63.8 ± 24.1	88.1 ± 10.4	0.001
∆ %SUVmax variation (12 weeks - baseline)	57.9 ± 31.5	90.7 ± 13.0	0.001

**Fig. 3:**
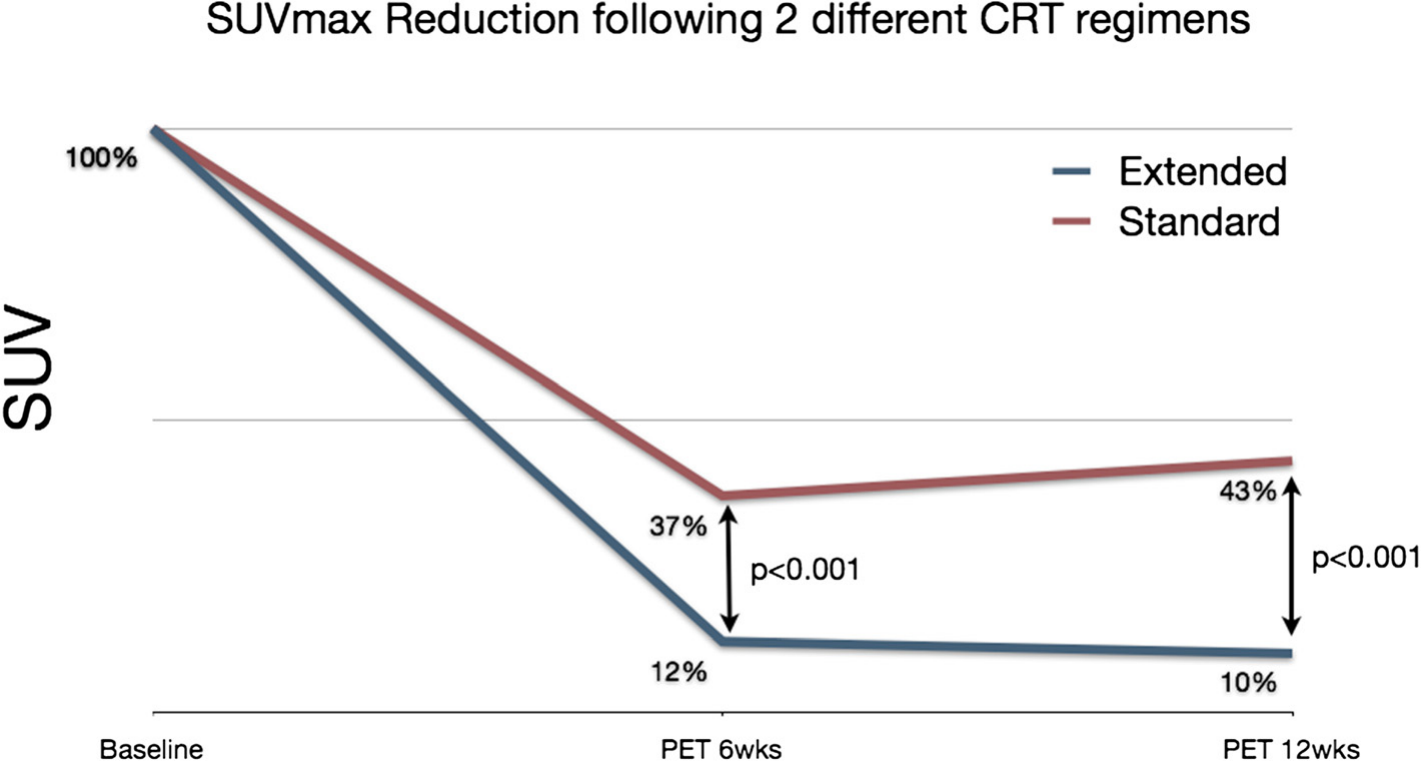
Variation in SUVmax between baseline and 6 and 12-week PET/CT; Patients undergoing consolidation CRT had significant increased variation between baseline and 6 or 12-week PET/CT (<0.001)

Curiously, percentage variation in SUVmax between 6 weeks and baseline was significantly higher among patients undergoing consolidation CRT. There was an 88 % reduction in SUVmax at 6 weeks among patients undergoing extended CRT as opposed to 63 % reduction among those undergoing standard CRT (*p* < 0.001). In addition, the percentage reduction at 12 weeks was even greater in the consolidation CRT group (90 % vs. 57 %; *p* < 0.001). (Fig. 3).

Between 6 and 12 weeks of RT completion, increases in SUVmax were observed in 46 patients out of 91 that performed all three PET/CTs (51 %) of the patients on the standard CRT arm. In contrast, increases in SUVmax were observed in only 2 patients (18 %) in the consolidation CRT group (*p* = 0.004).

## Discussion

Development of new alternative neoadjuvant regimens that could potentially increase complete tumor regression rates could have a major impact in the management and outcome of rectal cancer patients. Recently, an alternative CRT regimen incorporating additional cycles of 5FU-based chemotherapy (consolidation CRT) has resulted in significant increases in cCR rates ultimately avoiding surgery in more than half of patients with cT2/T3 rectal cancer [9]. In another recently reported phase II trial, the addition of multiple mFOLFOX6 cycles after CRT resulted in progressively higher complete pathological response rates after radical surgery [22].

It has been previously reported that a significant proportion of patients undergoing standard neoadjuvant CRT present an actual increase in SUVmax after 6 weeks from CRT completion, suggesting recuperation of metabolic activity of these tumors [19]. Patients that had an increase of metabolic activity after 6 weeks from standard CRT were found to have larger final tumor size. decreased rates of CR and decreased rates of near-complete responses. This could suggest that there was significant tumor repopulation at 6 weeks after standard CRT in a significant proportion of these patients [19]. In the present study, while 51 % of patients undergoing standard CRT had any increase in SUVmax between 6 and 12 weeks, only 18 % of patients undergoing extended CRT with consolidation chemotherapy were found to have this recuperation in metabolic activity. Altogether, these data suggest that after consolidation CRT, the average decrease in tumor metabolism is greater and tumor repopulation is less frequent when compared to standard CRT, at least between 6 and 12 weeks. Therefore, postponing assessment of tumor response after consolidation CRT until 12 weeks seems to be safer than doing so after standard CRT regimen. It may seem surprising that a rather modest increase in the amount of chemotherapy delivered after CRT may have resulted in such a significant reduction of tumor repopulation. However, one may have to consider that after a considerably high dose of radiation (54Gy), the presence of only residual microscopic foci could ultimately have been successfully eradicated by these additional cycles. In fact, the recently reported phase II trial suggests that the more additional chemotherapy, the higher chance of complete tumor regression and therefore, lesser chance of tumor cell repopulation [22]. Considering this latter study, there is a chance that not only the addition of oxaliplatin but also the use of more modern 5FU delivery options (oral or infusional) instead of bolus infusion used in our study, could contribute to increase response rates and block tumor cell repopulation.

One could argue that patients in the consolidation regimen had the last 2 PET/CTs performed while still on chemotherapy. This may have influenced the uptake of FDG by tumors cells resulting in decreased SUVmax values without representing true histological regression. This is the reason why it is recommended to perform FDG-PET imaging at least 2 weeks after completion of CRT for more reliable treatment monitoring. Still, the observed decreased SUVmax values after consolidation CRT did seem to correlate with final clinical outcomes by the overall cCR rates.

Finally, a longer follow-up with further sequential PET/CT imaging could have shown different outcomes. Ultimately, tumor repopulation after this extended CRT regimen may develop longer than 12 weeks. Therefore, longer follow-up could result in tumor recurrence (regrowths) after sufficient repopulation of cancer cells not detectable at 12 weeks due to the considerably small residual tumor burden. On the other hand, such study would require an excessive number of PET/CT imaging both in terms of cost and radiation exposure to these patients.

There have been a number of studies suggesting that a critical decrease in tumor metabolism that is associated with the development of a CR seems to be around 65 % after 6 weeks from CRT [13, 15–18]. Therefore, tumors that exhibit an average ≥65 % decrease in SUVmax after 6 weeks from CRT are more likely to develop the single most clinically relevant outcome after CRT for rectal cancer: complete response. In our previously reported study, the average reduction in SUVmax of rectal cancers after 12 weeks from standard CRT was 63 % [19]. In that study, there was no further reduction in average SUVmax between 6 and 12 weeks from RT completion when all patients are considered [19]. However, when only patients with a complete response are considered, there is further decrease in SUV values. The critical SUVmax reduction cutoff considered to be significant for CR development was ≥67 % at 6 weeks and ≥76 % at 12 weeks. This reduction was found to be an independent predictor of CR in that series [15]. In the present study, patients undergoing neoadjuvant consolidation CRT presented an average 88 % reduction in SUVmax between baseline and 6 week-PET/CT. This SUVmax reduction was significantly greater than the observed after standard CRT regimen (88 % vs. 63 %; *p* < 0.001). Considering that 65 % reduction was critical to the development of a CR, one could expect that an 88 % average reduction would result in a significant increase in the CR rate of patients undergoing this novel CRT regimen. In fact, complete response rates were also significantly higher after consolidation CRT as already suggested in previous reports and in this independent group of patients enrolled in this study (66 % vs 23 %; *p* < 0.001). In fact, even though 12 patients may constitute a rather small number of patients, the rates of initial CR are quite similar between the reported series so far [9, 20].

Limitations of the present study are considerable and may have accounted for some of the findings. First, the number of patients included in the extended consolidation CRT assessed by sequential PET/CT imaging is rather small and results warrant further investigation in a larger sample size. As a result of a limited sample size, even though there was no change in policy for the indication of neoadjuvant CRT between the two cohorts, patients in the extended CRT group had more earlier disease stage (cT2 vs. cT3) that could potentially have influenced CR rates. Still, the observed metabolism profile and percentage reduction in SUVmax may be possibly more related to sensitivity to CRT than actual T classification. Also, baseline SUVmax was significantly lower in the consolidation CRT group. There are conflictive data to suggest that baseline SUVmax is predictive of response to CRT in patients with rectal cancer. Higher SUV values have been associated with improved response in some studies. However, our data failed to correlate baseline SUV with response [15]. In addition, percentage variation rather that crude SUV values would potentially correct any bias in this direction. Ultimately, several features may influence individual SUVmax between tumors that are difficult to control for.

## Conclusions

Patients undergoing extended CRT with consolidation chemotherapy may develop more substantial reduction in tumor metabolism after 6 weeks from RT completion. This reduction in tumor metabolism may partially explain higher rates of complete regression after this treatment regimen. Also, the risk of developing tumor repopulation and increase in metabolism between 6 and 12 weeks is much lower after consolidation CRT. Therefore, assessment of tumor response may be safely postponed to 12 weeks in patients undergoing extended CRT (using consolidation chemotherapy) strategy.

## Abbreviations

CRT: neoadjuvant chemoradiation therapy
pCR: complete pathological response
CR: complete response
5FU: 5-Fluoracil
FDG: ^18^F-fluorodeoxyglucose
PET/CT: positron emission tomography/ computerized tomography
SUV: standard uptake values
RT: radiotherapy
DRE: digital rectal examination
ERUS: endorectal ultrasound
MR: magnetic resonance
cCR: complete clinical response
CT: computerized tomography
